# Purification and Biochemical Characterization of Polyphenol Oxidase from *Falcaria vulgaris* Bernh. †

**DOI:** 10.3390/molecules30244806

**Published:** 2025-12-17

**Authors:** Ceylan Buse Atlas Okut, Ayşe Türkhan

**Affiliations:** 1 Department of Biochemistry, Faculty of Science and Art, Iğdır University, 76000 Iğdır, Türkiye; buseatlas.1@hotmail.com; 2 Department of Chemistry and Chemical Processing Technologies, Vocational School of Technical Sciences, Iğdır University, 76000 Iğdır, Türkiye

**Keywords:** sickleweed (*Falcaria vulgaris* Bernh.), purification, polyphenol oxidase, characterization, inhibition

## Abstract

The polyphenol oxidase (PPO) enzyme leads to undesirable consequences by causing enzymatic browning during the processing of vegetables and fruits. As these browning reactions occur, many phenolic compounds of PPO can lead to significant changes in active metabolites due to substrate utilization. This may cause a loss of appearance and nutritional and commercial value of food. The sickleweed (*Falcaria vulgaris* Bernh.) plant studied in the current research is considered an edible and medicinal food. In the present research, polyphenol oxidase was purified 15.65-fold with a yield of 23.61% by affinity chromatography. The optimum pH and temperature for catechol, 4-methylcatechol, and 3,4-dihydroxyphenylpropionic acid substrates were determined in separate experiments. For all three substrates, the optimum pH was 7.0, while the optimum temperature was 20 °C. The catalytic efficiency ratio (*V*_max_/*K*_m_) was employed to assess the substrate specificity. Since the highest *V*_max_/*K*_m_ ratio reflects the greatest substrate affinity, 4-methylcatechol was identified as the substrate with the highest affinity for sickleweed PPO based on these values. pH stability and thermal stability were examined in the presence of 4-methylcatechol. The inhibitory effects of widely used antibrowning agents, sodium metabisulphite, citric acid, and ascorbic acid, on PPO activity were investigated. The results show that ascorbic acid was the most efficient inhibitor.

## 1. Introduction

*Falcaria vulgaris* is a fast-growing edible and medicinal plant from the Apiaceae (Umbelliferae) family, with an average height of 30 cm. In Iğdır province, it is cooked with yogurt or milk and bulgur, roasted with eggs and pickled. The plant’s leaves are called sickleweed and gazeyağı because they resemble a goose’s foot. Geographically, it is spread in America, Europe, Türkiye, Iran, the Caucasus, Central Asia, and Northwest Africa [1,2,3]. In Persian medicine, *Falcaria vulgaris* Bernh. is widely used to prevent, control, and treat many diseases [4]. Research has demonstrated that sickleweed has anti-inflammatory, antiviral, antibacterial, antifungal, and antioxidant effects [5,6]. The literature reports that sickleweed is used for medical purposes in the Iranian region to treat gastrointestinal [7] and skin diseases [5], fertility regulation [8], heart diseases [9], and as an anti-cancer agent [10]. *Falcaria vulgaris* Bernh. contains various phenolic constituents and flavonoids, which contribute significantly to the plant’s specific biological activities. Some environmental factors can easily affect the contents of these chemical components. These compounds found in plants may cause a browning reaction due to the activity of polyphenol oxidase during processing, impact, and storage [11].

Polyphenol oxidase (E.C.1.14.18.1) represents a copper-dependent metalloenzyme responsible for enzymatic browning during harvesting, storing, processing, and handling various plant materials. PPO is ubiquitously present in plants and other organisms [12,13]. PPO catalyzes two main reactions: (1) the hydroxylation of monophenols to form o-diphenols, and (2) the oxidation of o-diphenols to produce o-quinones. The products acquired at the end of the oxidation reactions are converted into brown, red, or black pigments by polymerization [14,15]. While these browning reactions occur, many phenolic compounds of PPO may cause significant changes in active metabolites due to substrate use. This may cause a loss of appearance and food’s nutritional and commercial value [13,16].

PPO, one of the enzymes involved in enzymatic browning, has been inactivated by inhibitors, and various studies have been carried out to control this process [17]. Diverse methods for the inhibition of polyphenol oxidase can be applied to prevent enzymatic browning. These methods include removing O_2_ or substrates from the medium, reducing the pH to two or more units less than the optimum pH, temperature deactivation of the enzyme, adding compounds that inhibit PPO, and using substances that prevent melanin production [18]. The most effective control method is inhibiting enzymatic browning reactions with chemical and natural antibrowning agents [19]. Many chemicals have been used to inhibit the polyphenol oxidase enzyme. A few of these inhibitors can be safely used in the food industry because the chemicals used to inhibit PPO activity in foods should be non-toxic and allergen-free and not alter the food’s structural properties, taste, and aroma. L-cysteine, ascorbic, colic, and citric acids, which prevent enzymatic browning, are inhibitors that can be safely used in the food industry [20]. Although sulfites and their derivatives can prevent enzymatic browning quite effectively, they adversely affect human health [17].

Several studies have researched the impacts of PPO, the causative agent of enzymatic browning, on postharvest and processed food quality in many fruits and vegetables such as *Agaricus bisporus* [16], purslane [21], potato (*Solanum tuberosum*) tubers [22], *Camellia sinensis* [23], rape flower [24], water yam (*Dioscorea alata*) [25], *Ocimum basilicum* L. [26] and edible yam (*Dioscorea opposita* Thunb.) [27]. The literature review shows that PPO from sickleweed has not been purified and characterized. This research aimed to purify, characterize, and inhibit PPO from sickleweed, which has both food and medicinal uses.

## 2. Results and Discussion

### 2.1. Purification of PPO from Sickleweed

**Figure 1:**
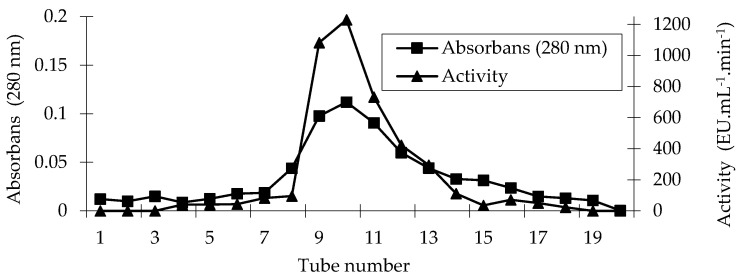
Affinity chromatography profile of the PPO purified from sickleweed (*Falcaria vulgaris* Bernh.).

PPO was purified from sickleweed (*Falcaria vulgaris* Bernh.) using an affinity column. A qualitative protein assay was performed on the eluates using the Walburg–Christian method [28] and activity determination was conducted using a 4-methylcatechol substrate (Figure 1). Quantitative protein determination in the crude enzyme extract and pure enzyme was performed following the Bradford method [29]. Table 1 contains purification data for PPO.

PPO from sickleweed was purified 15.65-fold with a 23.61% yield using the affinity column (Table 1). By utilizing the same affinity column, PPO was purified 13.9-fold from *Lactarius piperatus* [30], 11.2-fold from Cimin grape (*Vitis vinifera* spp., Cimin) [31], 43-fold from artichoke (*Cynara scolymus* L.) [32], 5.21-fold from unripe Japanese pear (*Pyrus pyrifolia* (Burm.) Nakai) fruit, and 9.77-fold from ripe ones [33].

### 2.2. Native Polyacrylamide Gel Electrophoresis and Sodium Dodecyl Sulfate Polyacrylamide Gel Electrophoresis

**Figure 2:**
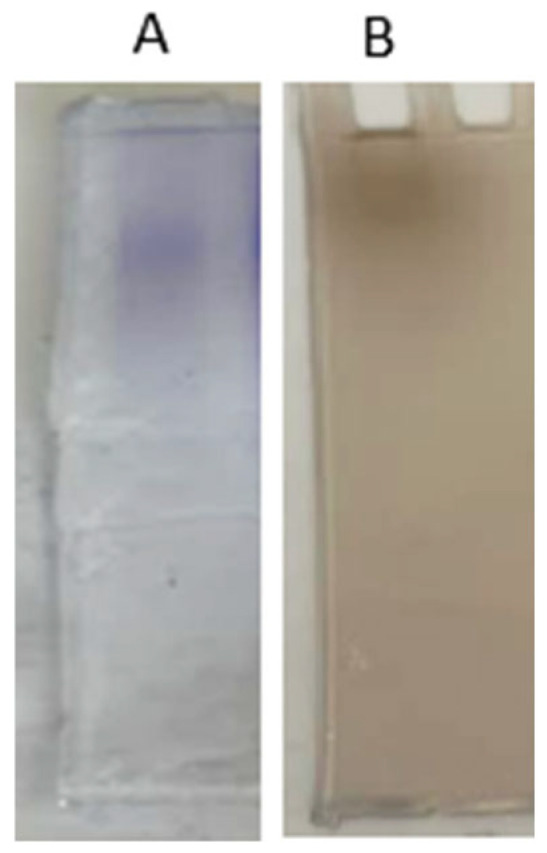
Native PAGE staining of the PPO purified from sickleweed (*Falcaria vulgaris* Bernh.) (**A**) Coomassie Brilliant Blue-R250 staining (**B**) 24 mM L-DOPA staining.

The purity of the PPO purified from sickleweed was determined by performing native polyacrylamide gel electrophoresis. The observation of a single band in the Coomassie Brilliant Blue-R250 staining of the resulting gel shows that the enzyme was purified (Figure 2A). Additionally, another gel prepared with the same properties was stained with Levodopa (L-DOPA) as a substrate staining solution, and a single band was obtained (Figure 2B). These results show that the enzyme was purified and that the purified enzyme was PPO (Figure 2). To demonstrate the purity of the PPO purified from different sources, native PAGE was carried out, the gel was stained with Coomassie Brilliant Blue-R250, and a single band was found. L-DOPA was also used for substrate staining, and a single band was found [15,34,35,36].

**Figure 3:**
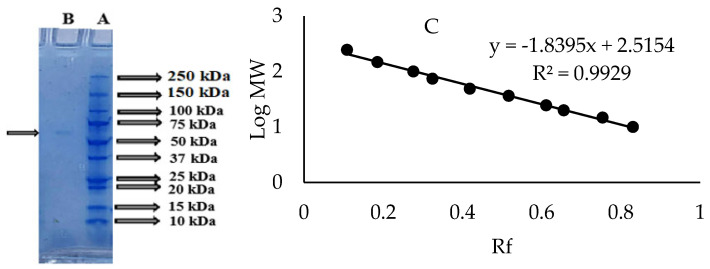
SDS-PAGE image (**A**) Protein standard (**B**) Coomassie Brilliant Blue-R250 staining of the PPO purified from sickleweed (*Falcaria vulgaris* Bernh.) (**C**) Log Mw-Rf graph.

The molecular weight of the PPO purified from sickleweed was found to be approximately 67.60 kDa using the Log Mw-Rf graph by SDS-PAGE (Figure 3). The literature review showed that the PPO purified from different sources had different molecular weights. The molecular weight of PPO was calculated as 65 kDa for mulberry (*Morus alba* L.) [37], 60 kDa for oil lettuce (*Lactuca sativa* var. *capitata* L.) [38], 72.44 kDa for plum (*Prunus domestica*) [39], 50 kDa for tea leaf (*Camellia sinensis*) [40] and 40 kDa for *Boletus erythropus* [14].

### 2.3. The Optimum pH

**Figure 4:**
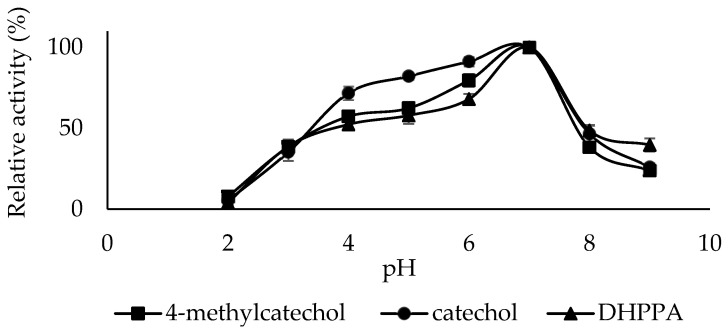
Optimum pH plots of PPO purified from sickleweed (*Falcaria vulgaris* Bernh.) using three substrates: catechol, 4-methylcatechol, and DHPPA. Experiments were performed in triplicate, and error bars represent standard deviations.

Enzymes affect the ionization of amino acids in the active site depending on the pH level of their medium. This changes the enzyme’s conformation and affects the substrate’s binding strength and affinity. Generally, the optimum pH of PPO varies depending on the substrates used and the enzyme source and extraction method [41]. The optimum pH of sickleweed PPO was 7.0 for catechol, 4-methylcatechol, and DHPPA substrates (Figure 4). For PPO from corn stover, the optimum pH was 8.0 for catechol and 6.0 for 4-methylcatechol [42], For LacPPO, the optimum pH was 5.0 for 4-methylcatechol and 7.0 for DHPPA [43]. For PPO from Kirmizi Kismis grape (*Vitis vinifera* L.), the optimum pH was 5.0 for the 4-methylcatechol substrate [44]. For PPO from *Cistanche deserticola*, the optimum pH was 7.0 for the catechol substrate [45].

### 2.4. Determination of the Optimum Temperature

**Figure 5:**
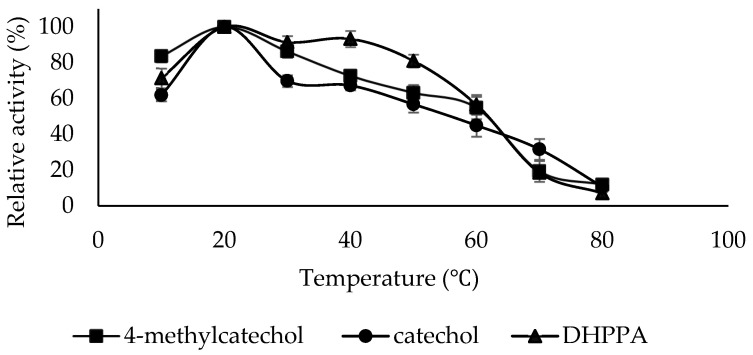
Optimum Temperature plots of PPO purified from sickleweed (*Falcaria vulgaris* Bernh.) using three substrates: catechol, 4-methylcatechol, and DHPPA. Experiments were performed in triplicate, and error bars represent standard deviations.

The optimum temperature for PPO activity can vary based on the enzyme’s source, the environmental conditions under which the source is cultivated, and the specific substrate employed [46]. It is, therefore, essential to research the optimum temperature for polyphenol oxidase enzymes isolated from new sources. The optimum temperature for PPO in the presence of 4-methylcatechol, catechol, and DHPPA substrates was found to be 20 °C (Figure 5). For LacPPO, the optimum temperature was 20 °C for 4-methylcatechol and 30 °C for DHPPA [43]. For PPO from Sarali plum, the optimum temperature was 20 °C for catechol and -4-methylcatechol [39]. For PPO from the persimmon fruit, the optimum temperature was 20 °C for catechol, 40 °C for 4-methylcatechol, and 60 °C for DHPPA [47].

### 2.5. K_m_ and V_max_

**Figure 6:**
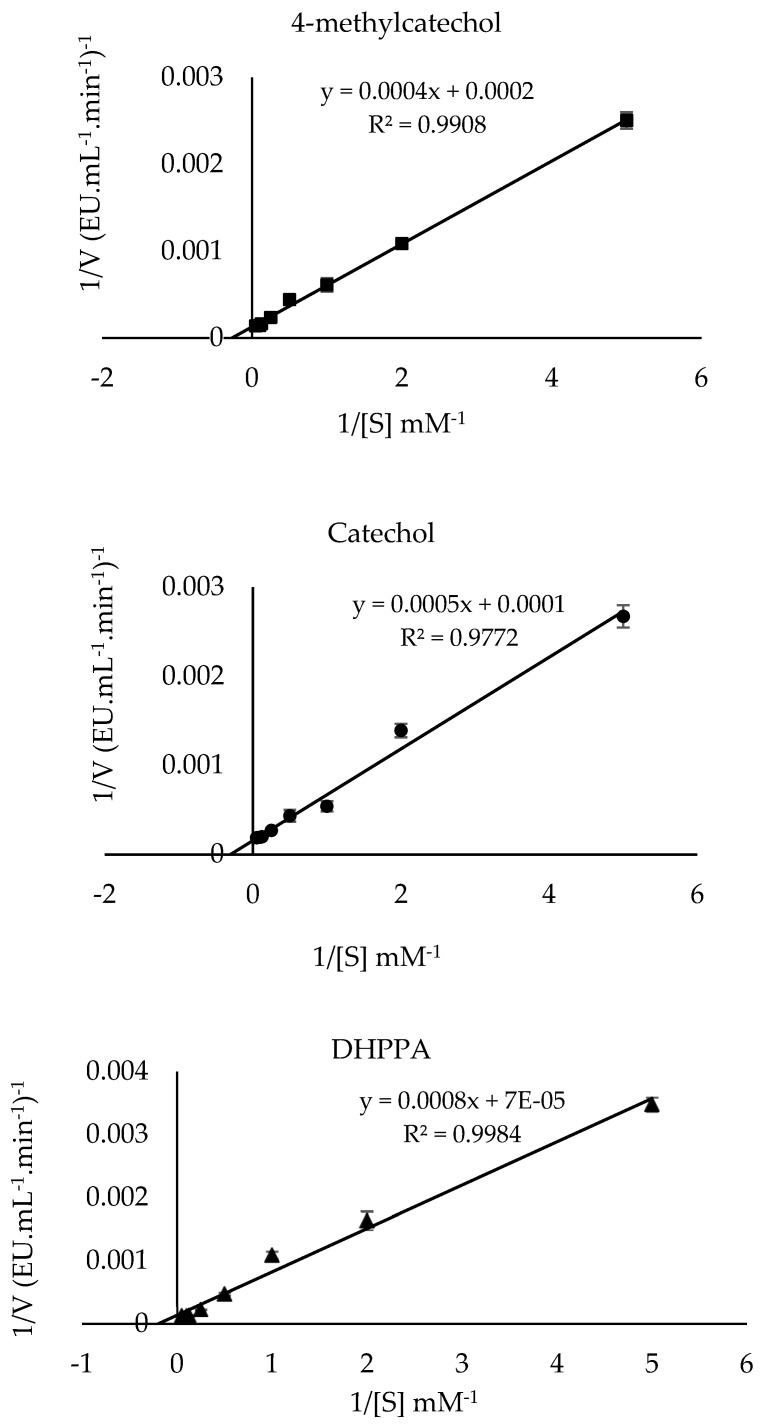
Lineweaver–Burk plots of PPO purified from sickleweed (*Falcaria vulgaris* Bernh.) using three substrates: 4-methylcatechol, catechol, and DHPPA. Experiments were performed in triplicate, and error bars represent standard deviations.

The Michaelis-Menten constant (*K*_m_) and the maximum velocity (*V*_max_) values of PPO in the presence of 4-methylcatechol, catechol, and DHPPA substrates were calculated by utilizing the Lineweaver–Burk plots. Table 2 lists the *K*_m_, *V*_max_, and *V*_max_/*K*_m_ values, and the results are compared with those of other studies in the literature. The *K*_m_ and *V*_max_ values of sickleweed PPO in the presence of 4-methylcatechol, catechol, and DHPPA were computed to be 2 mM and 500 EU·mL^−1^·min^−1^ (Figure 6), 5 mM and 10,000 EU·mL^−1^·min^−1^ (Figure 6), and 11.43 mM and 14,285.71 EU·mL^−1^·min^−1^ (Figure 6), respectively. This study used the catalytic efficiency ratio (*V*_max_/*K*_m_) to determine the substrates’ specificity [48]. The (*V*_max_/*K*_m_) ratios for catechol, 4-methylcatechol, and DHPPA substrates were calculated as 2000 (EU·mL^−1^·min^−1^), 2500 (EU·mL^−1^·min^−1^), and 1249.84 (EU·mL^−1^·min^−1^), respectively. The highest (*V*_max_/*K*_m_) value indicates the best substrate affinity. Among the substrates studied, sickleweed PPO had the highest (*V*_max_/*K*_m_) value for 4-methylcatechol (Table 2). The literature has reported that the wide range of *K*_m_ values of PPO originates from different source types, the use of different purification methods, substrates, and buffers [49].

### 2.6. pH Stability

Temperature and pH are two parameters that influence the enzyme’s catalytic activity. pH and temperature can vary in stability depending on the source of enzymes, the buffers, and the substrates used. These two parameters should work to control the enzyme’s activity [20,48]

**Figure 7:**
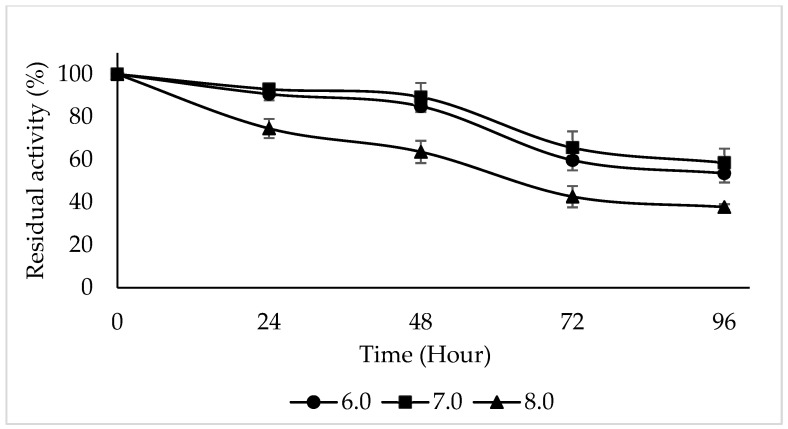
pH stability of PPO purified from sickleweed (*Falcaria vulgaris* Bernh.) was evaluated in the pH range 6.0–7.0–8.0 after incubation at 4 °C for 24, 48, 72, and 96 h using 4-methylcatechol as the substrate. Experiments were performed in triplicate, and error bars represent standard deviations.

The enzyme activity was maintained at different rates at pH 6.0, 7.0, and 8.0 for 24, 48, 72, and 96 h. After 24 h of incubation at 4 °C using 4-methylcatechol as a substrate, PPO activity was preserved over 90% at pH 6.0, 7.0, and 8.0. PPO activity was maintained at 53.56%, 58.51%, and 37.78% at pH 6.0, 7.0, and 8.0 for 96 h of incubation at 4 °C (Figure 7). PPO from the snake fruit was found to retain more than 80% of its initial activity in the pH range of 6.0–6.5 after 24 h of incubation at 4 °C and lost almost 50% of its initial activity above pH 7.5 [52]. Upon investigating the pH stability of PPO purified from *Macrolepiota gracilenta*, it was reported that after 5 days of incubation at 4 °C, PPO retained its activity by 62% at pH 5.0 and 42% at pH 7.0 and lost its activity at pH 8.0 [15]. The pH stability of Ispir sugar bean was examined in the range of 4.0–8.5 for 10 days, and it was found that the enzyme activity was highest at pH 6.0 and was maintained at different rates at other pH values [53].

### 2.7. Thermal Stability

**Figure 8:**
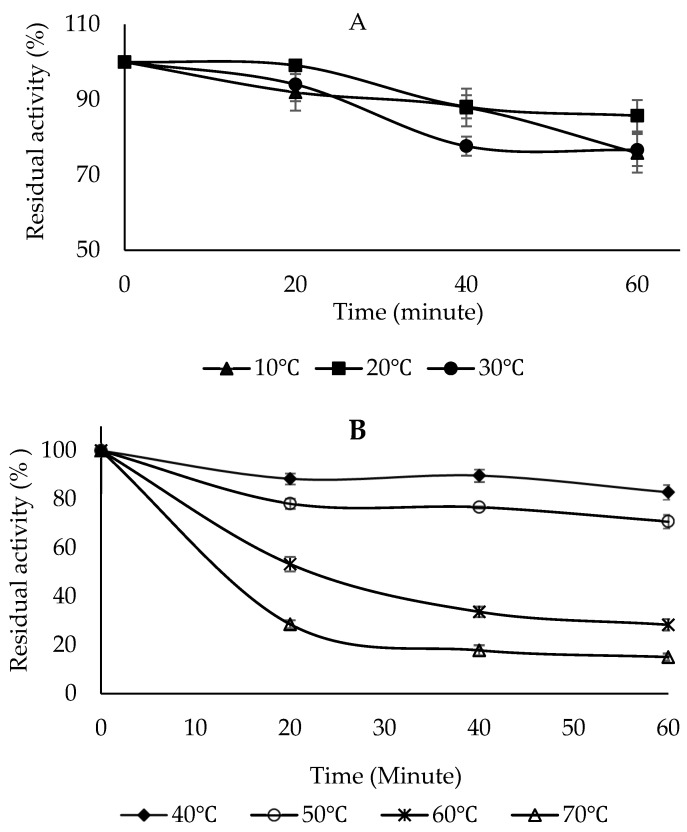
Temperature stability of PPO purified from sickleweed (*Falcaria vulgaris* Bernh.). (**A**) Enzyme activity after incubation at 10, 20, and 30 °C for 20, 40, and 60 min. (**B**) Enzyme activity after incubation at 40, 50, 60, and 70 °C for 20, 40, and 60 min. 4-Methylcatechol was used as the substrate. Experiments were conducted in triplicate, and error bars indicate standard deviations.

To determine the thermal stability of PPO, the enzyme solution was studied in the range of 10–70 °C in 10 °C increments for 20, 40, and 60 min using the 4-methylcatechol substrate (Figure 8A,B). The thermal stability results showed that the enzyme maintained its activity best at 20 °C after 60 min of incubation. At higher temperatures, the enzyme activity gradually decreased with increasing temperature and incubation time (Figure 8B). After 60 min of incubation, the enzyme activity was retained by 28.37% at 60 °C and 15.08% at 70 °C, respectively (Figure 8B). The thermal stability of Sarali plum (*Prunus domestica*) PPO was investigated in the presence of catechol and 4-methylcatechol, and it was found that the enzyme activity was maintained by 92% and 88% at 4 °C for 90 min, respectively. At 60 °C, the enzyme activity was preserved by 22% and 30.5% in the presence of catechol and 4-methylcatechol, respectively. At 70 °C, the activity was preserved by 6.6% and 5.1% at the end of 90 min, respectively [39]. The PPO obtained from the banana was thermally stable at 30 °C. Even at temperatures above 60 °C, PPO could retain some of its activity [54]. The PPO from green bean (*Phaseolus vulgaris* L.) was thermally stable between 0 and 40 °C for 30 min [55]. Medlar fruit PPO was moderately stable at the optimum temperature and up to 60 °C for 30 min. It was reported that enzyme activity decreased after 10 min of incubation at high temperatures, and this loss of activity could be due to deterioration in the enzyme’s secondary and tertiary structures [51].

### 2.8. Inhibition

**Figure 9:**
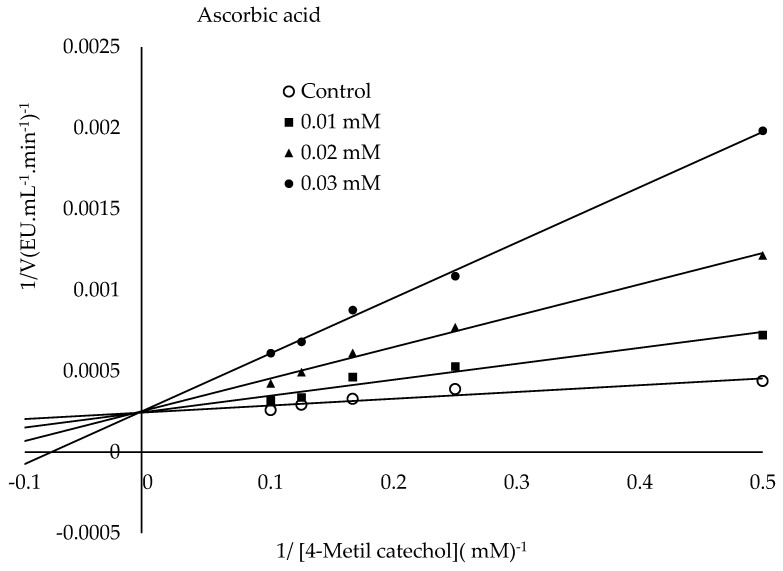

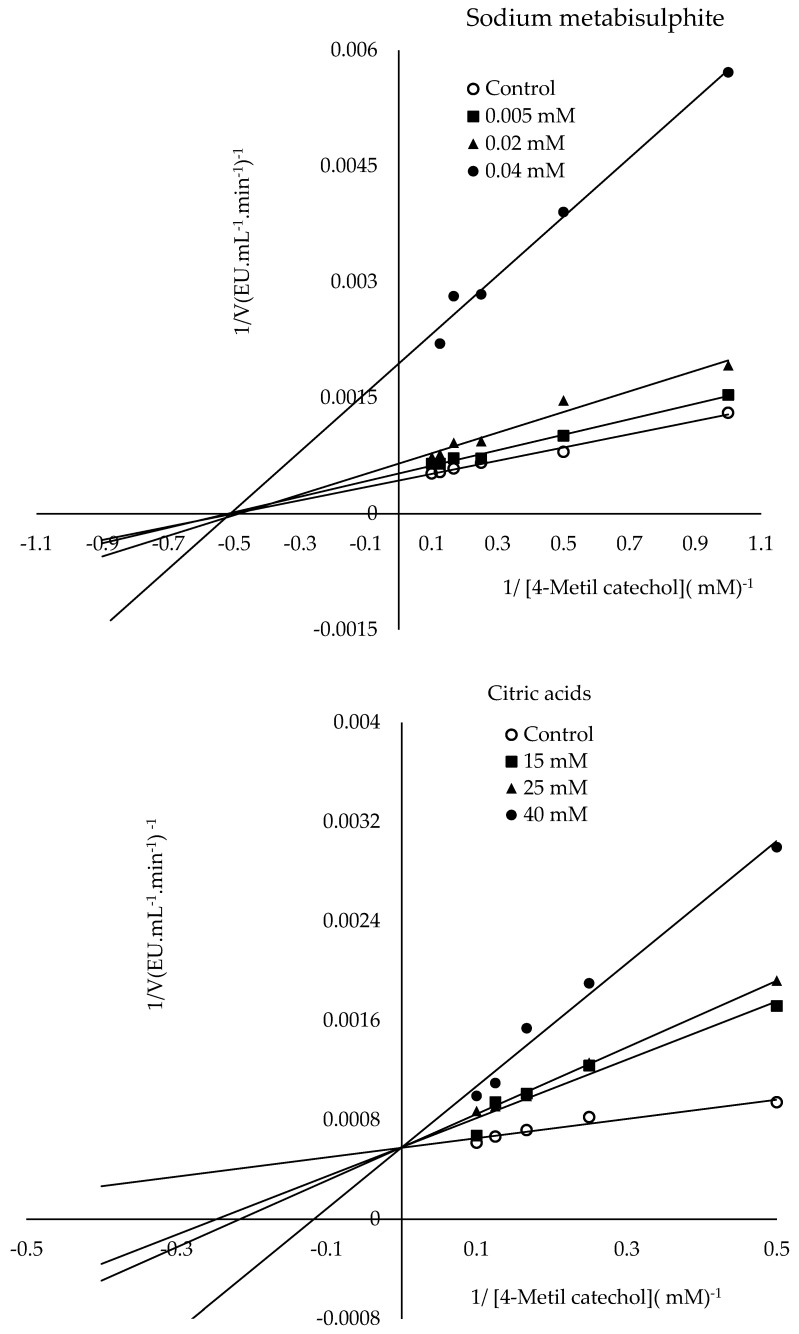
Lineweaver–Burk plots were drawn to determine the *K*_i_ constants of ascorbic acid, sodium metabisulfite, and citric acid in the presence of 4-methylcatechol as the substrate.

The mechanism of L-ascorbic acid inhibition is that PPO oxidizes phenolic substrates to o-quinones, whereas L-ascorbic acid converts the formed o-quinones back into phenolic compounds [56]. Additionally, ascorbic acid can cause PPO inhibition by reducing the pH value of the medium to which it is added or by capturing the oxygen in the medium necessary for the PPO reaction [57,58]. The literature has described the inhibitory mechanism of citric acid as follows. It causes PPO inhibition by lowering the pH value or chelating copper in the enzyme’s active site [59]. It has been suggested that SH groups display a strong affinity to copper, potentially displacing histidine residues coordinated to the copper in PPO’s active site or even causing the copper to be removed from the enzyme entirely. Furthermore, PPO inhibitors are categorized into two groups: those interacting with the copper binding site and those affecting the phenolic site. The category interacting with the copper site displays competitive inhibition. The other category exhibits non-competitive inhibition [40]. The present research found the IC_50_ values for ascorbic acid, sodium metabisulphite, and citric acid to be 0.026 mM, 0.027 mM, and 31.98 mM, respectively. The *K*_i_ constants and modes of inhibition were determined for the three inhibitors from the Lineweaver-Burk plots (Figure 9). The lowest *K*_i_ constant was observed for ascorbic acid (0.0101 mM), followed by sodium metabisulphite (0.0237 mM) and citric acid (8.73 mM) (Figure 9). The present research findings demonstrated that ascorbic and citric acids exhibited competitive inhibition, while sodium metabisulphite demonstrated non-competitive inhibition. Ascorbic acid showed the most potent inhibition among the inhibitors tested. Table 3 presents a comparison of this research with other studies in the literature. The literature has reported that the type of inhibition varies depending on the source of PPO, the substrate, and the inhibitor studied [60].

## 3. Materials and Methods

### 3.1. Materials and Chemicals

The plant used in this study, sickleweed (*Falcaria vulgaris* Bernh.), was obtained from a local market in Iğdır province, Turkey, in June 2024, and stored at −20 °C until use. The plant was identified by Prof. Dr. Ahmet Zafer TEL and deposited at the Iğdır University National Wildlife Museum (INWM) Herbarium under the accession number INWM00000236.

All chemicals and reagents employed in this study were of analytical grade. Sodium acetate (CH_3_COONa), potassium phosphate monobasic (KH_2_PO_4_), potassium phosphate dibasic (K_2_HPO_4_), tris base, glycine, 3-methyl-2-benzothiazolinone hydrazone (MBTH), HCl, cyanogen bromide-activated-Sepharose™ 4B, dimethylformamide (DMF), L-3,4-dihydroxyphenylalanine (L-DOPA), 4-methylcatechol, ascorbic acid, sodium metabisulfite, citric acid, catechol, Coomassie Brilliant Blue R-250 and DHPPA were purchased from Sigma Chem. Co. (St. Louis, MO, USA) and Merck A.G. (Darmstadt, Germany). Spectrophotometric measurements were carried out using a UV–Vis Spectrophotometer (Agilent Cary 60, Santa Clara, CA, USA). Native and SDS–PAGE electrophoresis experiments were performed using a Bio-Rad electrophoresis system (Hercules, CA, USA).

### 3.2. Methods

#### 3.2.1. Preparation of the Crude Enzyme Extract

Five grams were taken from the sickleweed plant, put into the mortar, and crushed. After several freeze–thaw cycles, 10 mL of 50 mM pH 5.0 acetate buffer containing 1% PEG was added. The mixture was thoroughly mixed, filtered, and centrifuged at 10,000 rpm for a period of 30 min, and a supernatant was utilized as crude enzyme extract [62].

#### 3.2.2. Determination of Polyphenol Oxidase Activity

PPO activity was determined by measuring the increase in absorbance at 496 nm for 4-methylcatechol and 500 nm for catechol and DHPPA with a UV–Vis spectrophotometer [63]. PPO activity was detected by the increment in absorbance in 1 min by adding 100 μL of the substrate (100 mM), 100 μL of 3-Methyl-2-benzothiazolinone hydrazone (MBTH, 10 mM), 20 μL of Dimethylformamide (DMF), 680 μL of 50 mM pH 5.0 acetate buffer, and finally 100 μL of the enzyme. The blank contains all solutions, excluding the enzyme. The enzyme unit is defined as the amount of the enzyme producing an absorbance increase of 0.001 per minute in 1 mL of the reaction mixture. The specific activity of PPO was defined as units per mg of protein [64].

#### 3.2.3. Purification of Polyphenol Oxidase from Sickleweed

The was synthesized following Arslan et al. (2004) [37]. The column was balanced with sodium acetate buffer (50 mM pH 5.0). The enzyme was applied to an affinity column. The column was washed with 50 mM pH 5.0 sodium acetate buffer with the objective of removing impurities. The PPO enzyme retained on the affinity column was collected in affinity column (Sepharose-4B-l-Tyr-p-amino benzoic acid) tubes of 2 mL each using 50 mM pH 8.0 phosphate buffer containing 1 M NaCl. Protein and activity determination was performed separately in the collected tubes.

#### 3.2.4. Native-PAGE and SDS-PAGE

A 5% loading gel and a 12% separating gel were used for native PAGE [65]. After the gels were ready, they were placed in the tank and loaded with proteins. The tank was placed in a container filled with ice to prevent protein denaturation. After the gel was run, it was removed from the tank and stained with a freshly prepared 24 mM L-DOPA substrate solution.

A 5% loading gel and a 12% separating gel were used for SDS-PAGE [65]. The proteins were subjected to the steps required for SDS-PAGE. At the end of the run, the gel was costained with Coomassie Brilliant Blue R-250. The bands were visualized when they became visible.

#### 3.2.5. Optimum pH

Glycine-HCI (pH 2.0–3.0), sodium acetate (pH 4.0–5.0), phosphate (pH 6.0–7.0), and tris-HCl (pH 8.0) buffers at a concentration of 50 mM were utilized to investigate the impact of pH on polyphenol oxidase activity [66]. The pH value at which polyphenol oxidase activity was highest was considered 100%. Other values were calculated as % relative activity over this value, and the pH-% relative activity graph was plotted.

#### 3.2.6. Optimum Temperature

To assess the impact of temperature on PPO activity, the enzyme was measured in a water bath at temperatures between 10 and 70 °C. The reaction medium containing the buffer (at optimum pH) and substrate were kept in the water bath at the specified temperatures for 10 min, following which enzyme activity was measured. The obtained data were analyzed and a plot of percent relative activity versus temperature was constructed [15].

#### 3.2.7. Kinetic Studies

To determine substrate specificity, catechol (0.2–20 mM), 4-methylcatechol (0.2–20 mM), and DHPPA (0.2–20 mM) concentrations were studied. Enzyme activity was carried out at optimum temperature and pH. The kinetic data were analyzed in terms of 1/activity (1/V) and 1/substrate concentration ([S]). The Michaelis-Menten constant (*K*_m_) and the maximum velocity (*V*_max_) were computed by plotting the Lineweaver-Burk graph. The catalytic efficiency ratio (*V*_max_/*K*_m_) was used as a criterion to evaluate substrate specificity [67].

#### 3.2.8. Thermal Stability

The thermal stability of the purified PPO was studied by keeping the enzyme for 20, 40, and 60 min in the temperature range of 10–70 °C using 4-methylcatechol as a substrate. At the end of the period, the enzyme was put in an ice container for 5 min and brought to room temperature. The activity of the enzyme was carried out under determined optimum conditions. The activity of the unincubated PPO was taken as 100%, the required calculations were performed, and the graph was plotted [43].

#### 3.2.9. pH Stability

Phosphate (pH 6.0–7.0, 50 mM) and 50 mM tris-HCl (pH 8.0) buffers were used to investigate the pH stability of PPO. The enzyme was mixed with the specified buffers at a 1:1 ratio and incubated at a temperature of 4 °C for 24, 48, 72, and 96 h. The activity of the enzyme was carried out under determined optimum conditions. The activity of the unincubated PPO was taken as 100%, the required calculations were performed, and the graph was plotted [51].

#### 3.2.10. Inhibition of PPO

The common inhibitors of PPO were preferred to study the inhibitory effects on PPO activity. For the 4-methylcatechol substrate, sodium metabisulphite (0.005–0.1 mM), citric acid (5–60 mM), and ascorbic acid (0.005–0.04 mM) inhibitors were utilized to determine enzyme activities under optimum conditions. The half maximal inhibitory concentration (IC_50_), which halves the enzyme activity, was calculated using the graph of percentage residual activity (%) versus inhibitor concentration (enzyme activity in the absence of inhibitor was accepted as one hundred percent). Then, enzyme activities were studied at five substrate concentrations and three inhibitor concentrations under optimum conditions. The Lineweaver-Burk graphs were drawn by calculating 1/V and 1/[S] values with the obtained data. From these graphs, inhibition constant (*K*_i_) values were calculated for each inhibitor, and the inhibition type was determined [67].

## 4. Conclusions

In the present research, PPO was purified from sickleweed (*Falcaria vulgaris* Bernh.) using affinity chromatography. Native and SDS-PAGE showed the enzyme’s purity. The optimum pH and temperature values for PPO were determined to be similar to those found in the literature. Substrate specificity was studied in the presence of catechol, 4-methylcatechol, and DHPPA substrates. By calculating the catalytic activity (*V*_max_/*K*_m_) values, it was determined that the enzyme had the highest affinity for 4-methylcatechol. IC_50_ and *K*_i_ values and types of inhibition were found for ascorbic acid, citric acid, and sodium metabisulfite. The best inhibitor was ascorbic acid. Sickleweed is an edible and medicinal plant. Hence it is important to control PPO activity to prevent browning during sickleweed processing and storage. In addition, the purification and inhibition of PPO from the sickleweed plant may contribute to preserving and increasing the effectiveness of the plant’s biological and medicinal activities by preventing the oxidation of phenolic compounds.

## Figures and Tables

**Table 1:** Table for the purification of sickleweed PPO.

Purification Steps	Volume (mL)	Total Activity	Total Protein (mg)	Specific Activity (U/mg Protein)	Yield (%)	Purification Fold
Crude ezyme etract	4	10,417.33	5395.03	1.93	100	1
Affinity chromatography	2	2460	81.41	30.22	23.61	15.65

**Table 2:** Comparison of *K*_m_ and *V*_max_ values from the current work with other studies in the literature.

Source of Enzyme	Substrate	*K*_m_ (mM)	*V*_max_ (EU·mL^−1^·min^−1^ or μM/min *)	*V*_max_/*K*_m_ (EU·mL^−1^·min^−1^·mM^−1^ or min^−1^ *)	Refs.
Chinese parsley (*Coriandrum sativum*)	Catechol	31	2000	64.52	[50]
	4-Methylcatechol	37.43	1428.57	38.17	
	DHPPA	-	-	-	
Sarali plum (*Prunus domestica*)	Catechol	1.16 ± 0.12	914.14 ± 83.45	790.91 ± 37.34	[39]
	4-Methylcatechol	4.75 ± 0.66	2333.33 ± 288.68	492.06 ± 13.75	
	DHPPA	-	-	-	
Butter lettuce (*Lactuca sativa* var. *capitata* L.)	Catechol	3.20 ± 0.01	4081 ± 8		[38]
	4-Methyl catechol	1.00 ± 0.09	5405 ± 4		
	DHPPA	-	-		
*Laccaria laccata*	Catechol	0.25	-	-	[43]
	4-Methylcatechol	0.40	-	-	
	DHPPA	-	-	-	
Medlar PPO	Catechol	5.7	88.0 *	0.0154 *	[51]
	4-Methylcatechol	7.5	130 *	0.0173 *	
	DHPPA	1.9	7.2 *	0.0038 *	
Persimmon fruits (*Diospyros kaki* L., Ebenaceae)	Catechol	12.4	55.2 *	0.0044 *	[47]
	4-Methylcatechol	14.6	49.5 *	0.0034 *	
	DHPPA	12.8	17.2 *	0.0013 *	
*Falcaria vulgaris* Berhn.	Catechol	5	10,000	2000	The study presented
	4-Methylcatechol	2	5000	2500	
	DHPPA	11.43	14,285.71	1249.84	

The values marked with an asterisk (*) in the table are expressed in the units given in parentheses in the header.

**Table 3:** Comparison of IC_50_ and *K*_i_ values from this study with those from other studies in the literature.

Enzyme Source	Substrate	Inhibitor	IC_50_ (mM)	*K*_i_ (mM)	İnhibition Type	Refs.
Broccoli (*Brassica oleracea* var. botrytis italica) florets	4-Methylcatechol	Ascorbic acid	-	8.8 × 10^−2^	Noncompetitive	[61]
		Sodium metabisulfite	-	-	-	
		Citric acid	-	7.4 × 10^−2^	Noncompetitive	
Tea leaf (*Camellia sinensis*)	catechol	Ascorbic acid	69.3 × 10^−3^	32.67 × 10^−3^ ± 0.016	Competitive	[40]
		Sodium metabisulfite	21.65 × 10^−3^	19.30 × 10^−3^ ± 0.005	Noncompetitive	
		Citric acid	-	-	-	
Purslane	catechol	Ascorbic acid	-	0.36	Noncompetitive	[21]
		Sodium metabisulfite	-	-	-	
		Citric acid	-	4.47	Uncompetitive	
Sarali plum	4-Methylcatechol	Ascorbic acid	0.038 ± 0.001	0.0095	Competitive	[39]
		Sodium metabisulfite	-	-	-	
		Citric acid	-	-	-	
*Falcaria vulgaris* Bernh.	4-Methylcatechol	Ascorbic acid	0.026	0.0101	Competitive	This study
		Sodium metabisulfite	0.027	0.0237	Noncompetitive	
		Citric acid	31.98	8.37	Competitive	

## Data Availability

The data that support the findings of this study are available from the corresponding author upon reasonable request.

## Abbreviations

PPO	Polyphenol oxidase
*V*_max_/*K*_m_	Catalytic efficiency ratio
L-DOPA	Levodopa
*DHPPA*	3,4-Dihydroxyphenylpropionic acid
*K*_m_	Michaelis-Menten constant
*V*_max_	Maximum velocity
IC_50_	Half maximal inhibitory concentration
*K*_i_	Inhibition constant
Native-PAGE	Native Polyacrylamide Gel Electrophoresis
SDS-PAGE	Sodium Dodecyl Sulfate Polyacrylamide Gel Electrophoresis
MBTH	3-Methyl-2-benzothiazolinone hydrazone
DMF	Dimethylformamide
Affinity column	Sepharose-4B-l-Tyr-p-amino benzoic acid
